# Supplementary material for: Yiai Fuzheng decoction inhibits triple-negative breast cancer by remodeling the immune microenvironment
Source: Front Immunol. 2025 Sep 30;16:1615631. doi: 10.3389/fimmu.2025.1615631 (PMC12518410; doi:10.3389/fimmu.2025.1615631)
Supplement: Supplementary file 2 [file DataSheet1.zip › Supplementary File 2/Treatment/D_vs_H/07_Pathway_Analysis/Enrichment_Analysis/predicted/Network.pdf]

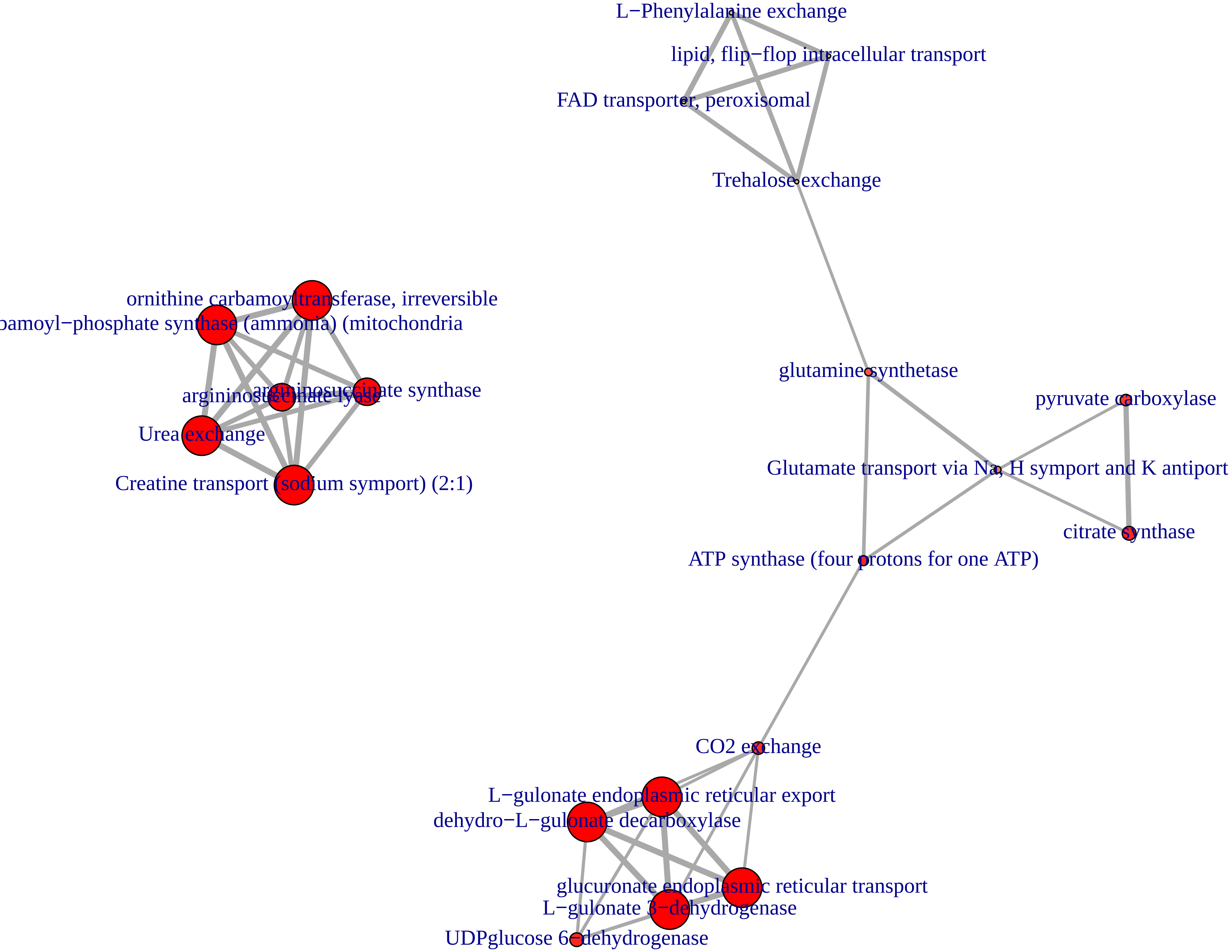
L−Phenylalanine exchange
lipid, flip−flop intracellular transport
FAD transporter, peroxisomal
Trehalose exchange
ornithine carbamoyltransferase, irreversible
bamoyl−phosphate synthase (ammonia) (mitochondria
argininosuccinate lyase
argininosuccinate synthase
Urea exchange
Creatine transport (sodium symport) (2:1)
glutamine synthetase
pyruvate carboxylase
Glutamate transport via Na, H symport and K antiport
citrate synthase
ATP synthase (four protons for one ATP)
CO2 exchange
L−gulonate endoplasmic reticular export
dehydro−L−gulonate decarboxylase
glucuronate endoplasmic reticular transport
L−gulonate 3−dehydrogenase
UDPglucose 6−dehydrogenase
